# Inadequate Prenatal Visit and Home Delivery as Determinants of Perinatal Outcomes: Does Parity Matter?

**DOI:** 10.1155/2019/9024258

**Published:** 2019-04-10

**Authors:** Nigus Bililign Yimer, Zelalem Tenaw, Kalkidan Solomon, Tesfahun Mulatu

**Affiliations:** ^1^Department of Midwifery, Woldia University, Ethiopia; ^2^Department of Midwifery, Hawassa University, Ethiopia; ^3^Department of Public Health, Wolkite University, Ethiopia; ^4^Department of Public Health, Woldia University, Ethiopia

## Abstract

**Background:**

Adverse perinatal outcomes are still high in developing countries. Contradicting evidences were reported about the effect of parity on adverse perinatal outcomes. The aim of this study was to compare perinatal outcomes in grand multiparous and low multiparity women in Hawassa University Comprehensive Specialized Hospital and Adare General Hospital of Ethiopia.

**Methods:**

Comparative cross-sectional study design was employed to include 461 mothers from February to June 2018. Data were collected by structured questionnaire using interview and from patient charts. Data were entered using EPI-DATA version 4.4.2.0. Descriptive statistics and logistic regression analyses were computed using STATA version 14 computer software.

**Results:**

Of all study participants, 24.9% (95% Confidence interval: 21.1%-29.1%) had at least one adverse perinatal outcome. Stillbirth (38.9), low Apgar score (51.9%), and congenital malformation (3.70%) were frequently occurred complications in grand multiparas compared to low multiparous women. Nevertheless, meconium aspiration, need for resuscitation, and macrosomia were higher in low multiparous women (9.84%, 14.75%, and 57.38%, respectively). Less than four prenatal visits (AOR: 1.74; 95% CI: 1.04, 2.92) and previous home delivery (AOR: 1.87; 95% CI: 1.04, 3.33) were independent predictors of adverse perinatal outcomes. However, parity did not show statistically significant difference in perinatal outcomes.

**Conclusion:**

This finding underscores the fact that frequency of antenatal care and place of delivery are significant predictors of perinatal outcomes. However, parity did not show statistically significant difference in perinatal outcomes. Women empowerment, promoting health facility delivery, and early, comprehensive antenatal care are needed.

## 1. Introduction

Every year, more than two million stillbirths occur, a third of them in sub-Saharan Africa [1]. Ninety-nine percent of neonatal deaths occurred in low- and middle-income countries, mainly from preventable causes [2]. Worldwide, infant deaths are attributed to multiple economic, maternal, psychosocial, and health behavior factors [3].

One cohort finding showed that admission of neonatal intensive care unit was significant among newborns born to grand multiparous women [4]. Grand multiparity (≥5 live births/stillbirths) was also associated with low Apgar score [5]. Similarly, adverse outcomes were seen among high parity women [6]. On the contrary, in Uganda, stillbirth risks decreased with increasing parity (≥5) [7]. A cohort study in the same country revealed absence of difference in fetal outcomes between grand (5-9 deliveries) and low multiparous (para 2-4) women [8].

In Brazil, factors related to quality of prenatal care were associated with high chance of death in preterm infants [9]. Multiple deprivation and poor psychosocial support were determinants of late prenatal presentation and adverse fetal outcomes [10]. A study in low-resource settings revealed that women with less antenatal care and delivered without skilled birth attendant were more likely to have a stillbirth [11].

In China, hypothyroidism was significantly related to intrauterine growth restriction and low birth weight [12]. Additionally, a Zambian study reported that low birth weight was associated with placental abruption, multiple gestation, and preterm delivery [13]. Low socioeconomic status and female sex had also positive association with low birth weight [14]. A cross-sectional study in northern Ethiopia reported the significant association of parity, lack of antenatal care, and male sex with congenital anomalies [15].

Inadequate engagement with prenatal care is associated with unfavorable birth outcomes [10]. In Ethiopia, there is paucity of comparative researches on perinatal outcomes across parity groups. The finding of this may serve as a baseline to undertake large studies to show the effect of parity on birth outcomes. Hence, this study aimed to compare perinatal outcomes in multiparous women and determine independent factors associated with adverse perinatal outcomes in Hawassa Hospitals.

## 2. Methods

### 2.1. Study Setting, Population, and Design

Comparative cross-sectional study was deployed from February 1 to June 30, 2018, in Hawassa University Teaching Hospital and Adare General Hospital. In obstetrics and gynecology unit of Hawassa University Teaching Hospital, there are 9 obstetricians and gynecologists and 54 midwives. Similarly, one obstetrician and gynecologist, four Integrated Emergency Surgery and Obstetrics (IESO) professionals, 15 midwives, three nurses, and two public health officers attend obstetric ward of Adare General Hospital. All multiparous mothers who gave birth in the study areas during the study period were the source population of this study. All multiparous, laboring mothers were the study population. All multiparas with a single fetus/neonate at a gestational age of ≥28 weeks were included in the study. Multiparas who were not able to communicate or seriously ill mothers were excluded from the study.

### 2.2. Sample Size Determination

The sample size was computed using double population proportion formula from Epi-Info version 7.2.2.6 computer software. The following assumptions were made: power of the study (1-*β*) to be 80%, 95% confidence interval (CI), the estimated unexposed-to-exposed ratio to be 2:1, and percent of outcome among nonexposed group & odds ratio of previous studies [5, 16, 17] were used. Thus, adding 10% nonresponse rate, the final sample size was 471 (157 grand multiparas and 314 low multiparas).

### 2.3. Sampling Procedure

Study subjects were identified during time of admission to labor ward. When eligible mothers were identified after delivery, admission and registration books as well as patient charts were checked for prepartal conditions. The total average number of deliveries was estimated to be 762 per month in the two study hospitals. Sample size was allocated proportionally to study sites based on their monthly flow of clients for delivery. Thus, a sample of 255 (85 GM & 170 LM) and 216 (72 GM & 144 LM) were allocated to Hawassa University Teaching Hospital and Adare General Hospital, respectively.

### 2.4. Study Variables

The main outcome/dependent variable was adverse perinatal outcome. Independent/exposure variables were sociodemographic variables (age, parity, income, education level, etc.) and antenatal profile and obstetric characteristics (gestational age at first booking, hypertension, diabetes mellitus, previous history of preterm delivery, intrauterine fetal death, previous caesarean scar, number of prenatal visits, previous home delivery, etc.).

### 2.5. Operational Definitions

*Perinatal Outcome*. In this study, perinatal outcome was at least one adverse outcome of the fetus/newborn (stillbirth, mal-presentation, macrosomia, low Apgar score, etc.) between 28 weeks of gestation and discharge from the hospital. In this study, grand multiparity and low multiparity were defined as ≥5 and 2-4 births after the age of viability, respectively [18].

### 2.6. Data Collection Tool and Procedure

Data were collected by six trained diploma-holder midwives in the two study sites. One Bachelor of Science holder midwife was recruited as supervisor at each study area. The investigator trained data collectors and supervisors for three days about the tool and data collection procedures. The data were collected by face-to-face interview and review of clinical documents.

The standard questionnaire has three sections. The first section was demographic characteristics of the study subjects like age and parity. The second section was obstetric characteristics of respondents such as hypertension and diabetes in current pregnancy, previous history of stillbirth and preterm delivery. The final section of the tool consisted of perinatal outcomes (macrosomia, low birth weight, congenital malformations, low Apgar score, etc.).

For mothers who had normal delivery, data were collected 1-2 hours after delivery. Mothers who had caesarean or complicated vaginal delivery waited until they fully awake to respond the questions.

### 2.7. Data Quality Control and Analysis

Pretest was done on 5% of the sample size in one hospital other than the study areas (Yirgalem Hospital). Another reproductive health specialist checked validity of the tool. The final pretested and checked structured tool was used for the data collection.

On each day of data collection, the supervisors and principal investigator checked the completeness of the data. Incomplete questionnaires were discarded. Data were coded and entered to Epi-Data version 4.4.2.0 and then exported to STATA version 14.1 computer software for analysis. Univariate analysis and cross-tabulation of variables were done for outcome and independent variables. The chi-square test X^2^ was used to test for overall significance. Variables with a* p* value ≤0.25 were included in the multivariable logistic regression analyses. Statistically significant variables were declared at* p* value less than 0.05.

### 2.8. Ethical Considerations

Institutional Review Board (IRB) of Hawassa University College of Medicine and Health Sciences approved this study. Support letter was written to the study hospitals from Department of Midwifery. Written informed consent was obtained from study participants after the data collectors explained the objective of the study. Confidentiality was also assured by anonymizing names of respondents.

## 3. Results

### 3.1. Sociodemographic Characteristics of Respondents

The mean age (±SD) of the participants was 28.7 (±4.7) and ranged from 18 to 48 years. Majority of the respondents who develop adverse perinatal outcomes (23.99%) were within the age group of 21-34 years. Forty-seven (32.64%) rural residents had adverse perinatal outcome, whereas majority (78.55%) urban residents had no complications (chi^2^* p*=0.010). From the total study participants, only six mothers were household heads (single, widowed, and divorced). Out of the total illiterate participants, more than one-third had adverse perinatal outcomes than 120 (76.43%) primary school attendees without complications (chi^2^* p*=0.001) [Table 1].

### 3.2. Obstetric Profile of Participants

The mean birth weight (±SD) of newborns was 2994.80 (±601.87) and 3214.98 (±564.60) grams for grand multiparas and low multiparas, respectively. In the grand multiparous women, more than one-third (34.39%) participants had adverse perinatal outcomes than 60 (20.07%) in the low multiparous counterparts (chi^2^ p=0.001). Adverse perinatal outcomes were common in women having less four prenatal visits than mothers who had 4 times or more visits (39.23% vs. 20.65%). Additionally, 46 (36.22%) respondents who had home delivery prior to the current one develop perinatal complications than only 20.66% of mothers who gave birth at health institutions (chi^2^* p*=0.001). Injectable and implants were the most frequently used contraceptives in respondents' life time. Perinatal complications were reported as higher in preterm labor (63.64%) and postterm pregnancy (62.50%) than in term gestations (chi^2^* p*=0.001). Higher proportions of male fetuses develop perinatal complications than females [Table 2].

### 3.3. Adverse Perinatal Outcomes

**Figure 1:**
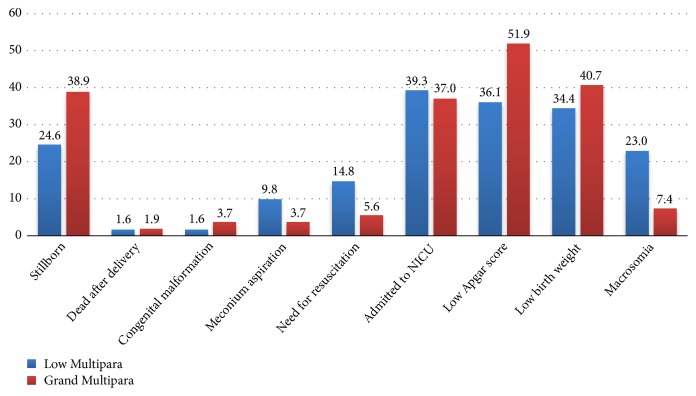
Adverse perinatal outcomes in Hawassa University Comprehensive Specialized Hospital & Adare General Hospital, 2018. Pearson's* p* value=0.19.

The prevalence of adverse perinatal outcome was 24.9% (95% CI: 21.1%, 29.1%). Stillbirth (38.9%), low Apgar score (51.9%), and congenital malformation (3.7%) were frequently occurred complications in grand multiparas than in low multiparous women. Nevertheless, meconium aspiration, need for resuscitation, and macrosomia were higher in low multiparous women (9.84%, 14.75%, and 57.38%, respectively) [Figure 1].

### 3.4. Predictors of Adverse Perinatal Outcome

In the univariable logistic regression analysis, candidate variables in the chi-square test were computed with the outcome variable; adverse perinatal outcome (yes/no). Then, variables with* p* value less than 0.25 were candidates for the final model (see Table 3).

In the multivariable logistic regression model, number of Antenatal Care (ANC) visits and place of last delivery were found to be independent predictors of adverse perinatal outcome. Mothers who had less than four prenatal visits were at risk for perinatal complications by 74% (AOR: 1.74; 95% CI: 1.04, 2.92). Similarly, the odds of adverse perinatal outcomes increased by 87% for mothers who had previous home delivery. However, parity did not show statistically significant association with the outcome variable [Table 3].

## 4. Discussion

This finding revealed that many adverse perinatal complications (stillbirth, congenital malformations, low Apgar score, and low birth weight) were reported to be higher in grand multiparous women. Previous home delivery and number of prenatal visits were significantly associated with adverse perinatal outcomes. However, parity did not show significant difference in low and grand multiparous women.

In this study, place of delivery was found to be a significant predictor of adverse perinatal outcomes. Mothers who gave birth at home during their last delivery were 87% more likely to develop adverse perinatal outcomes in current pregnancy. A large population-based study in low-and middle-income countries showed that women who had no skilled birth attendant during delivery were at significant risk of stillbirth [11]. A cross-sectional study in China showed that neonatal death was significantly lower in women who gave birth in country-level hospitals [2]. Another retrospective cohort study showed that newborns born to rural mothers were at risk of severe neonatal morbidity, being born preterm, having low Apgar score, and being large for gestational age [19]. In the United Kingdom, direct associations were noted between socioeconomic factors to utilize health services and adverse perinatal outcomes [20]. In Southern Ethiopia, stillbirth and neonatal mortality rates were higher in areas where institutional delivery was very low [21]. This may imply the need of promoting institutional delivery service utilization. Ally with traditional birth attendants may be also important to increase utilization of delivery at health facilities. Community mobilization and participatory approaches to address cultural factors that affect use of health facilities might have paramount benefits.

The present study showed the significant association of perinatal complications and frequency of prenatal visits. Mothers who had suboptimal prenatal visits (1 to 3 times) were at higher risk of perinatal complications. Similarly, a finding from national maternal survey of Ghana reported decreased odds of stillbirth in women who complete the recommended four prenatal visits [1]. Furthermore, a retrospective evidence from Tanzania showed increased odds of low birth weight in women having less than four ANC visits [22]. A cross-sectional evidence from China reported significant association between neonatal death and lack of prenatal care in the first trimester [2]. A population-based multicountry study revealed that stillbirth rate was significantly higher in women with less access to antenatal care [11]. Another prospective study from Mekelle, Ethiopia, reported that congenital malformations were significantly associated with lack of antenatal care visit [15]. As evidenced by one cross-sectional study, newborns born to Mexican women with inadequate prenatal care were at increased risk for low birth weight [23]. A longitudinal study in Bahir Dar, Ethiopia, showed that access to quality ANC was a key strategy to improve birth weight [24]. This indicates that adequate and timely use of prenatal care may help to prevent perinatal complications. Identifying the barriers, which affect frequency of ANC visits (like transportation, health professionals approach, and mothers' attitude), might be important to implement strategies. This finding might also be an input to implement the new WHO recommendation on frequency of prenatal visits. The organization recommended eight or more contacts for antenatal care to reduce perinatal deaths by 8 per 1000 births [25].

In the current study, there was no statistically significant difference in perinatal outcomes between grand multiparous and low multiparous women. Nevertheless, stillbirth, low birth weight, and low Apgar score were higher in grand multiparous women than in low multiparas. On the other hand, macrosomia was reported to be higher in the low multiparous group. One study reported the insignificant increase of neonatal complications in grand multiparous women [4]. As parity increases, a decline in risk of stillbirth was noted in rural Uganda [7]. A cohort study in Oman reported the protective effect of grand multiparity for low birth weight [26]. Other studies also reported the insignificant effect of parity on perinatal outcomes [8, 27, 28]. On the contrary, grand multiparity was found to be significantly associated with poor fetal outcomes [5, 16, 29]. These differences might be due to differences in study design, sample size, possible confounders, and other methodological issues. Additionally, accessible and quality antenatal care differences in study subjects could explain this. Thus, universal and meticulous prenatal care for all mothers and special care for high-risk groups may prevent adverse perinatal outcomes.

This study has certain limitations. Because of cross-sectional design's nature, we could not show the direction of association. Recall bias on previous obstetric characteristics and incompleteness of patient chart are also limitations of this study.

## 5. Conclusion

The present study showed that adverse perinatal outcome was independently associated with previous home delivery and frequency of ANC visits in the current pregnancy. However, parity did not show statistically significant difference in perinatal outcomes. Promotion of adequate prenatal care and utilization of health facility delivery is needed.

## Figures and Tables

**Table 1:** Distribution of sociodemographic characteristics by perinatal outcomes in HUCSH & AGH, Southern Ethiopia, September 2018.

Variables	Adverse perinatal outcomes, n (%)		*P* value
	Yes (115)	No (346)	
Maternal age			
≤20	2 (15.38)	11 (84.62)	0.324*∗*
21-34	89 (23.99)	282 (76.01)	
>34	24 (31.17)	53 (68.83)	

Residence			
Rural	47 (32.64)	97 (67.36)	0.010
Urban	68 (21.45)	249 (78.55)	

Religion			
Protestant	71 (26.59)	196 (73.41)	0.341*∗*
Orthodox	18 (18.56)	79 (81.44)	
Muslim	26 (27.37)	69 (72.63)	
Others	0 (0.00)	2 (100.00)	

Region			
SNNPR	64 (21.84)	229 (78.16)	0.040*∗*
Amhara	10 (22.22)	35 (77.78)	
Oromo	45 (34.45)	78 (65.55)	
Others	0 (0.00)	4 (100.00)	

Marital status			
Married	115 (25.27)	340 (74.73)	0.344*∗*
Others	0 (0.00)	6 (100.00)	

Mothers' education			
None	40 (36.36)	70 (63.64)	0.001*∗*
Read and write only	2 (5.13)	37 (94.87)	
Primary	37 (23.57)	120 (76.43)	
Secondary	20 (26.32)	56 (73.68)	
College and above	16 (20.25)	63 (79.75)	

Mothers' occupation			
Housewife	83 (28.23)	211 (71.77)	0.096
Government employee	15 (18.75)	65 (81.25)	
Self-employed	17 (19.54)	70 (80.46)	

Income			
Lower tertile	50 (30.67)	113 (69.33)	0.105
Middle tertile	34 (22.52)	117 (77.48)	
Upper tertile	31 (21.09)	116 (78.91)	

Husband education			
None	22 (37.29)	37 (62.71)	0.001
Read and write only	5 (10.64)	42 (89.36)	
Primary	30 (24.00)	95 (76.00)	
Secondary	33 (34.74)	62 (65.26)	
College and above	25 (18.52)	110 (81.48)	

Husband occupation			
Farmer	57 (34.13)	110 (65.87)	0.003
Government employee	28 (20.29)	110 (79.71)	
Self-employed	30 (19.23)	126 (80.77)	

*∗*Fisher's exact test; AGH: Adare General Hospital; HUCSH: Hawassa University Comprehensive Specialized Hospital; SNNPR: Southern Nations Nationalities and Peoples Region.

**Table 2:** Obstetric characteristics of respondents by perinatal outcomes in HUCSH & AGH, Southern Ethiopia, September 2018.

Variables	Adverse perinatal outcomes, n (%)		*P* value
	Yes (115)	No (346)	
Gravidity			
2-4	60 (19.93)	241 (80.07)	0.001
>4	55 (34.38)	105 (65.63)	

Parity			
Low multipara	61 (20.07)	243 (79.93)	0.001
Grand multipara	54 (34.39)	103 (65.61)	

Number of live births			
<5	75 (22.73)	255 (77.27)	0.081
≥5	40 (30.53)	91 (69.47)	

Past obstetric complications			
Yes	46 (28.57)	115 (71.43)	0.188
No	69 (23.00)	231 (77.00)	

Type of complications			
Abortion	18 (23.08)	60 (76.92)	0.028
IUFD	21 (42.86)	28 (57.14)	
Preterm delivery	2 (50.00)	2 (50.00)	
Instrumental delivery	1 (33.33)	2 (66.67)	
Cesarean section	10 (29.41)	24 (70.59)	
Others^+^	9 (42.86)	12 (57.14)	

Previous medical illness			
Yes	12 (36.36)	21 (63.64)	0.116
No	103 (24.07)	325 (75.93)	

Type of medical illnesses			
Hypertension	3 (21.43)	11 (78.57)	0.367*∗*
Diabetes mellitus	1 (33.33)	2 (66.67)	
Cardiac disease	1 (33.33)	2 (66.67)	
Others^++^	7 (53.85)	6 (46.15)	

ANC visit			
Yes	108 (26.60)	298 (73.40)	0.026
No	7 (12.73)	48 (87.27)	

GA at first booking			
≤16 weeks	34 (25.37)	100 (74.63)	0.694
>16 weeks	74 (27.21)	198 (72.79)	

Number of ANC visits			
1-3	51 (39.23)	79 (60.77)	0.001
≥4	57 (20.65)	219 (79.35)	

Place of delivery			
Home	46 (36.22)	81 (63.78)	0.001
Health Institutions	69 (20.66)	265 (79.34)	

Mode of delivery (before this birth)			
Vaginal	108 (26.28)	303 (73.72)	0.058
Cesarean section	7 (14.00)	43 (86.00)	

Distance from nearest health facility			
<15 minutes	16 (26.67)	44 (73.33)	0.780
15-30 minutes	26 (27.08)	70 (72.92)	
>30 minutes	73 (23.93)	232 (76.07)	

Contraceptive use			
Yes	77 (25.50)	225 (74.50)	0.706
No	38 (23.90)	121 (76.10)	

Type of contraception			
Injectable	51 (24.64)	156 (75.36)	0.723*∗*
Implants	11 (26.83)	30 (73.17)	
OCPs	10 (24.39)	31 (75.61)	
IUCD	4 (44.44)	5 (55.56)	
Natural method	1 (25.00)	3 (75.00)	

Planned pregnancy			
Yes	79 (23.87)	252 (76.13)	0.393
No	36 (27.69)	94 (72.31)	

GA for this birth			
Preterm	14 (63.64)	8 (36.36)	0.001
Term	37 (15.16)	207 (84.84)	
Post term	10 (62.50)	6 (37.50)	

Newborn sex			
Male	70 (28.93)	172 (71.07)	0.03
Female	45 (20.55)	174 (79.45)	

AGH: Adare General Hospital; HUCSH: Hawassa University Comprehensive Specialized Hospital;^+^early neonatal death, infant death, congenital malformation, ectopic pregnancy; ^++^hyperthyroidism, Deep Vein Thrombosis, acute abdomen, syphilis, Retroviral infection; *∗*Fisher's exact test; ANC: Antenatal Care; IUFD: intrauterine fetal demise; OCPs: oral contraceptive pills; IUCD: intrauterine contraceptive device; GA: gestational age.

**Table 3:** Univariable and multivariable logistic regression analysis of variables with adverse perinatal outcome in HUCSH & AGH, Southern Ethiopia 2018.

Variables	Adverse perinatal outcomes		COR (95% CI)	AOR (95% CI)	*P* value
	Yes	No			
Residence					
Rural	47 (32.64)	97 (67.36)	1.77 (1.14, 2.75)	1.32 (0.72, 2.41)	0.36
Urban	68 (21.45)	249 (78.55)	1	1	

Mothers' occupation					
Housewife	83 (28.23)	211 (71.77)	1.61 (0.89, 2.91)	1.22 (0.60, 2.44)	0.57
Government employee	15 (18.75)	65 (81.25)	0.95 (0.43, 2.05)	1.18 (0.49, 2.80)	0.70
Self-employed	17 (19.54)	70 (80.46)	1	1	

Income					
Lower tertile	50 (30.67)	113 (69.33)	1.65 (0.98, 2.77)	0.90 (0.45, 1.79)	0.76
Middle tertile	34 (22.52)	117 (77.48)	1.08 (0.62, 1.88)	0.79 (0.43, 1.47)	0.47
Upper tertile	31 (21.09)	116 (78.91)	1	1	

Husband occupation					
Farmer	57 (34.13)	110 (65.87)	2.17 (1.30, 3.62)	1.46 (0.73, 2.92)	0.28
Government employee	28 (20.29)	110 (79.71)	1.06 (0.60, 1.89)	1.15 (0.61, 1.47)	0.65
Self-employed	30 (19.23)	126 (80.77)	1	1	

Parity					
Low multipara	61 (20.07)	243 (79.93)	1	1	0.46
Grand multipara	54 (34.39)	103 (65.61)	2.08 (1.35, 3.21)	1.23 (0.70, 2.15)	

Previous medical illness					
Yes	12 (36.36)	21 (63.64)	1.80 (0.85, 3.79)	1.17 (0.49, 2.82)	0.71
No	103 (24.07)	325 (75.93)	1		

Number of ANC visits					
1-3	51 (39.23)	79 (60.77)	2.48 (1.57, 3.91)	**1.74 (1.04, 2.92)** **∗**	**0.03**
≥4	57 (20.65)	219 (79.35)	1	1	

Place of delivery					
Home	46 (36.22)	81 (63.78)	2.18 (1.39, 3.41)	**1.87 (1.04, 3.33)** **∗**	**0.03**
HI	69 (20.66)	265 (79.34)	1	1	

Mode of delivery (before this birth)					
Vaginal	108 (26.28)	303 (73.72)	1	1	0.15
Cesarean section	7 (14.00)	43 (86.00)	0.45 (0.19, 1.04)	0.50 (0.19, 1.28)	

Newborn sex					
Male	70 (28.93)	172 (71.07)	1.57 (1.02, 2.41)	1.32 (0.81, 2.13)	0.25
Female	45 (20.55)	174 (79.45)	1	1	

AGH: Adare General Hospital; HUCSH: Hawassa University Comprehensive Specialized Hospital; ANC: Antenatal Care Visits; AOR: adjusted odds ratio; COR: crude odds ratio; CI: confidence interval; HI: health institution; *∗*statistically significant at *p* value<0.05; 1 referent variable; Hosmer and Lemeshow goodness-of-fit= 0.24.

## Data Availability

The datasets used in this study are available from the corresponding author upon reasonable request.
