# Corrigendum to “Inadequate Prenatal Visit and Home Delivery as Determinants of Perinatal Outcomes: Does Parity Matter?”

**DOI:** 10.1155/2019/9161294

**Published:** 2019-08-04

**Authors:** Nigus Bililign Yimer, Zelalem Tenaw, Kalkidan Solomon, Tesfahun Mulatu, Abel Gedefaw

**Affiliations:** ^1^Department of Midwifery, Woldia University, Ethiopia; ^2^Department of Midwifery, Hawassa University, Ethiopia; ^3^Department of Public Health, Wolkite University, Ethiopia; ^4^Department of Public Health, Woldia University, Ethiopia; ^5^Department of Obstetrics and Gynecology, Hawassa University, Ethiopia

In the article titled “Inadequate Prenatal Visit and Home Delivery as Determinants of Perinatal Outcomes: Does Parity Matter?” [1], Dr. Abel Gedefaw was missing from the authors' list. Dr. Abel Gedefaw contributed to the proposal development and the article's draft. The corrected authors' list is shown above.
